# Outcomes of SBRT for lung oligo-recurrence of non-small cell lung cancer: a retrospective analysis

**DOI:** 10.1093/jrr/rrab118

**Published:** 2021-12-28

**Authors:** Qingren Lin, Ning Zhou, Xiang Zhu, Juan Lin, Jun Fang, Feiying Gu, Xiaojiang Sun, Yuezhen Wang

**Affiliations:** Department of Radiation Oncology, Cancer Hospital of the University of Chinese Academy of Sciences (Zhejiang Cancer Hospital), Institute of Cancer and Basic Medicine, Chinese Academy of Sciences, 1 Banshan Dong Road, Hangzhou, 310022, People’s Republic of China; Department of Radiation Oncology, Cancer Hospital of the University of Chinese Academy of Sciences (Zhejiang Cancer Hospital), Institute of Cancer and Basic Medicine, Chinese Academy of Sciences, 1 Banshan Dong Road, Hangzhou, 310022, People’s Republic of China; Department of Radiation Oncology, Cancer Hospital of the University of Chinese Academy of Sciences (Zhejiang Cancer Hospital), Institute of Cancer and Basic Medicine, Chinese Academy of Sciences, 1 Banshan Dong Road, Hangzhou, 310022, People’s Republic of China; Department of Radiation Oncology, Cancer Hospital of the University of Chinese Academy of Sciences (Zhejiang Cancer Hospital), Institute of Cancer and Basic Medicine, Chinese Academy of Sciences, 1 Banshan Dong Road, Hangzhou, 310022, People’s Republic of China; Department of Radiation Oncology, Cancer Hospital of the University of Chinese Academy of Sciences (Zhejiang Cancer Hospital), Institute of Cancer and Basic Medicine, Chinese Academy of Sciences, 1 Banshan Dong Road, Hangzhou, 310022, People’s Republic of China; Department of Radiation Oncology, Cancer Hospital of the University of Chinese Academy of Sciences (Zhejiang Cancer Hospital), Institute of Cancer and Basic Medicine, Chinese Academy of Sciences, 1 Banshan Dong Road, Hangzhou, 310022, People’s Republic of China; Department of Radiation Oncology, Cancer Hospital of the University of Chinese Academy of Sciences (Zhejiang Cancer Hospital), Institute of Cancer and Basic Medicine, Chinese Academy of Sciences, 1 Banshan Dong Road, Hangzhou, 310022, People’s Republic of China; Department of Radiation Oncology, Cancer Hospital of the University of Chinese Academy of Sciences (Zhejiang Cancer Hospital), Institute of Cancer and Basic Medicine, Chinese Academy of Sciences, 1 Banshan Dong Road, Hangzhou, 310022, People’s Republic of China

**Keywords:** oligo-recurrence, non-small cell lung cancer (NSCLC), lung metastases, stereotactic body radiotherapy (SBRT)

## Abstract

The benefit of local ablative therapy (LAT) for oligo-recurrence has been investigated and integrated into the treatment framework. In recent decades, stereotactic body radiation therapy (SBRT) has been increasingly used to eliminate metastasis owing to its high rate of local control and low toxicity. This study aimed to investigate the outcomes of SBRT for patients with lung oligo-recurrence of non-small cell lung cancer (NSCLC) from our therapeutic center. Patients with lung oligo-recurrence of NSCLC treated with SBRT between December 2011 and October 2018 at Cancer Hospital of the University of Chinese Academy of Sciences (Zhejiang Cancer Hospital) were reviewed. The characteristics, treatment-related outcomes, and toxicities of the patients were analyzed. Univariable and multivariable Cox regression were performed to identify the factors associated with survival. A total of 50 patients with lung oligo-recurrence of NSCLC were enrolled. The median follow-up period was 23.6 months. The 3-year local progression-free survival (LPFS), progression-free survival (PFS) and overall survival (OS) after SBRT were 80.2%, 21.9% and 45.3%, respectively. Patients in the subgroup with LAT to all residual diseases showed significantly improved OS and PFS. No treatment-related death occurred after SBRT. SBRT is a feasible option to treat patients with lung oligo-recurrence of NSCLC, with high rates of local control and low toxicity. LAT to all residual diseases was associated with better survival outcomes. Future prospective randomized clinical trials should evaluate SBRT strategies for such patients.

## INTRODUCTION

Despite major advances in therapeutic strategies in the last few decades, non-small cell lung cancer (NSCLC) is still the leading cause of cancer-related deaths worldwide [1]. The majority of patients with NSCLC might develop distant metastases throughout the course of the disease, and recurrence in distant organs is common even after definitive treatment [2]. In a subset of patients, the pattern of recurrence is limited to a few sites (≤5) with well-controlled primary cancer after initial therapy. The clinical phenomenon was initially proposed as ‘oligo-recurrence’ by Niibe, spurring the development of algorithms that integrate local metastases-directed therapy into systemic therapy [3]. Clinical evidence in recent years supported that local ablative therapies (LATs) were associated with improved treatment outcomes in a similar oligometastatic state [4, 5].

The lungs are common sites for metastatic recurrence from NSCLC. For patients with lung oligo-recurrence, pulmonary metastasectomy is the primary treatment option, and it yields good results with regard to local control and survival [6]. However, many patients with lung oligo-recurrence are unwilling or unable to tolerate the invasive treatment. Over the past decade, stereotactic body radiation therapy (SBRT) has become an alternative treatment for patients with early-stage and metastatic NSCLC owing to its safety and efficacy [7, 8]. In this study, we retrospectively investigated the safety and efficacy of SBRT for lung oligo-recurrence of NSCLC and aimed to analyze the characteristics of the patients and their survival outcomes.

## METHODS

### Patient eligibility

Patients with lung oligo-recurrence of NSCLC who were treated with SBRT between December 2011 and October 2018 at Cancer Hospital of the University of Chinese Academy of Sciences (Zhejiang Cancer Hospital) were eligible to participate in the study. The study protocol was approved by the medical ethics committee at our hospital (No.IRB-2021-86). The inclusion criteria were as follows: controlled primary tumor sites (any-stage NSCLC) without progression after initial therapy (including surgery/radiotherapy/chemotherapy/combined therapy), ≤3 metachronous lung metastases at the time of SBRT, and no other active sites of distant metastasis. Patients whose histopathology results were not available were clinically confirmed as having developed recurrence mainly based on the following factors: a treatment-free interval of more than 6 months, dynamic changes observed on radiological examination, and positive accumulation of tracer on fluorodeoxyglucose-positron emission tomography (FDG-PET) [9, 10]. To differentiate lung oligo-recurrence from second primary lung cancer, we used the Martini and Melamed criteria in histology [11]. Patients with metachronous solid or subsolid pulmonary nodules on computed tomography (CT) suspected as second primary lung cancer without being histologically proven were excluded. The patients were offered multidisciplinary consultations before SBRT.

Before SBRT, some patients were treated with LAT for the primary tumor, lymph nodes and metastatic sites. The method of LAT included surgery, definitive external beam radiation therapy (EBRT), both, or radiofrequency ablation; the method was determined according to the patients’ preference, performance status and treatment toxicities. Accepted definitive methods of EBRT included conventional fractionation radiotherapy (≥60 Gy in 2-Gy fractions), bone metastasis radiotherapy and whole-brain radiotherapy (30 Gy in 3-Gy fractions). The patients were divided into all-LAT and partial-LAT subgroups based on whether they were treated with LAT to all residual diseases or not.

### Treatment

Patients were immobilized in thermoplastic masks or vacuum cushions and underwent a 4-dimensional CT scan with 3-mm slice thickness. All the patients breathed freely during the simulation. The scan recorded the patients’ tumor motion related to respiration. The data of the respiratory phase and tumor were delivered to RayStation 4,5,1 system (RaySearch Laboratories AB, Stockholm, Sweden). Gross tumor volume (GTV) was drawn mainly based on the CT and FDG-PET CT findings in each respiratory phase. The internal target volume (ITV) was integrated from the GTVs. The planning target volume (PTV) was created from the ITV plus a 5-mm margin and 1.0 cm in the craniocaudal direction. The organs at risk, including the lungs, heart, spinal cord, trachea, esophagus and brachial plexus, were contoured according to several multicenter trials [12]. The treatment plan was generated in RayStation 4,5,1 system by using the method of conventional 6–12 beams or a volumetric modulated arc. The radiation therapy was performed with 6MV X-ray beams from Varian Trilogy-SN5387 linear accelerator (Varian Medical Systems Inc., Palo Alto, CA, USA) using non-coplanar or coplanar static ports. The highest isodose line of the prescribed dose was required to cover >95% of PTV and 100% of the ITV. The treatment schedule of multiple lung metastases is based on several factors, including the location and size of the lesions, the PTV relationship of the lesions and dose–volume constraints of the risk organs.

**Table 1:** Patient characteristics and details of local ablative therapy

Characteristic	Patient No. (%)
Age (y) <65 ≥65	27 (54) 23 (46)
Gender Female Male	15 (30) 35 (70)
ECOG PS 0–1 2	43 (86) 7 (14)
Histology Adenocarcinoma Squamous cell carcinoma Other NSCLCs	27 (54) 18 (36) 5 (10)
EGFR mutations Yes No	16 (32) 34 (68)
Stage IA IB IIA IIB IIIA IIIB IV	6 (12) 5 (10) 5 (10) 6 (12) 13 (26) 1 (2) 14 (28)
Diameter of lung metastases <2 cm ≥2 cm	35 (70) 15 (30)
Number of metastases 1 sites 2 sites 3 sites 5 sites	25 (50) 21 (42) 3 (6) 1 (2)
Previous metastatic organs Brain Lung Bone Adrenal glands Liver Chest wall Axillary lymph nodes Skin	5 (10) 3 (6) 4 (8) 3 (6) 2 (4) 4 (8) 1 (2) 1 (2)
Combined with chemotherapy or TKI Yes No	18 (36) 32 (64)
LAT for primary tumor site Surgery EBRT	27 (54) 9 (18)
LAT for primary metastasis sites	
Brain Whole-brain irradiation Surgery + whole-brain irradiation Stereotactic radiosurgery	1 (2) 2 (4) 1 (2)
Bone EBRT (30 Gy)	1 (2)
Liver Radiofrequency ablation	2 (4)
Lung metastasis SBRT	3 (6)
Adrenal EBRT (45 Gy)	2 (4)
LAT All-LAT Partial-LAT	34 (68) 16 (32)

LAT, local ablative therapy; ECOG, Eastern Cooperative Oncology Group; PS, performance status; NSCLC, non-small cell lung cancer; EGFR, epidermal growth factor receptor; TKI, tyrosine kinase inhibitor; EBRT, external beam radiation therapy; SBRT, stereotactic body radiation therapy

### Follow-up

Patients were followed up every 3 months, 6 months, 1 year and annually thereafter. A routine chest CT scan was essential during each follow-up. Additional imaging modalities including FDG-PET CT scans, bone scintigraphy, abdominal CT scans and brain CT or magnetic resonance imaging (MRI) were required to confirm suspected disease relapse. Dynamic growth of the treated site and the development of a new lesion next to PTV in the same lobe after SBRT was defined as local progression (LP). Similarly, a progression of the hilum, mediastinum, or supraclavicular fossa was defined as regional progression (RP). Distant metastasis progression (DMP) was defined as progression in a different lobe or other distant organs. Progression-free survival (PFS) was calculated from the time of diagnosis to the date of tumor progression (including LP, RP and DMP) or death. LPFS, RPFS and DMPFS were calculated from the time of diagnosis to the date of local, regional and DMP or death, respectively. Overall survival (OS) was the time between the day when oligo-recurrence was diagnosed and the event of death or the last follow-up. In addition, treatment-related toxicity was routinely evaluated according to the National Cancer Institute’s Common Terminology Criteria for Adverse Events (NCI CTCAE, version 4.0).

### Statistical analysis

Treatment outcomes were evaluated using Kaplan–Meier analysis of LP, RP, DMP, PFS and OS. The median follow-up duration was estimated using the reverse Kaplan–Meier method. Prognostic factors were analyzed using univariate and multivariate Cox regression analysis. A *P-*value less than 0.05 was considered statistically significant. All analyses were performed using IBM SPSS 21.0 software (IBM Corp., Armonk, NY, USA).

## RESULTS

### Patient and treatment characteristics

A total of 50 patients received SBRT for lung oligo-recurrence of NSCLC. The median interval between the initial treatment and the diagnosis of lung oligo-recurrence was 19.7 (range 6.1–101.93) months. In total, 25 (50%) patients just had one metastasis in the lungs, and 25 (50%) patients presented more than one distant metastases involving the lung, adrenal gland, brain and bone, chest wall, axillary lymph nodes and skin. Except for the lung metastases, which were active before SBRT, the other synchronous/metachronous oligometastatic sites were under control.

For the primary lung tumor site, 36 (72%) patients received LAT, namely 27 patients who underwent surgery and nine patients who received EBRT. With regard to the other primary distant metastasis sites, four (8%) patients received LAT to brain metastasis; one (2%) patient, to bone metastasis; two (4%) patients, to the liver; three (6%) patients, to the lung; and two (4%) patients, to the adrenal gland. Among them, 16 (32%) patients received LAT to either the primary lung tumor site or oligometastatic sites (partial-LAT subgroup) and 34 (68%) patients received LAT to both the primary lung tumor site and all oligometastatic sites (all-LAT subgroup). The patient characteristics and the details of LAT are summarized in Table 1.

For lung oligo-recurrence, all patients were treated with sufficiently high doses in such a way that the biologically effective dose (BED) was not less than 100 Gy. The total radiation dose of 50–70 Gy was delivered in different fractionation schemes (range, 4–10 fractions) (Table 2). During the SBRT period, 10 (20%) patients concurrently received chemotherapy (Paclitaxel, Docetaxel, Navelbine, Pemetrexed and Gemcitabine) and eight (16%) received oral EGFR-TKI (Erlotinib, Gefitinib, Icotinib and Osimertinib).

**Table 2:** Distribution of BED, fractions and doses

Prescribed total dose	No. of fractions	BED	Patient No. (%)
48	4	105.6	1 (2)
50	5	100	40 (80)
50	4	112.5	4 (8)
56	7	108.6	1 (2)
60	8	105	3 (6)
70	10	119	1 (2)

BED, biologically equivalent dose; SBRT, stereotactic body radiation therapy

### Survival

**Fig. 1:**
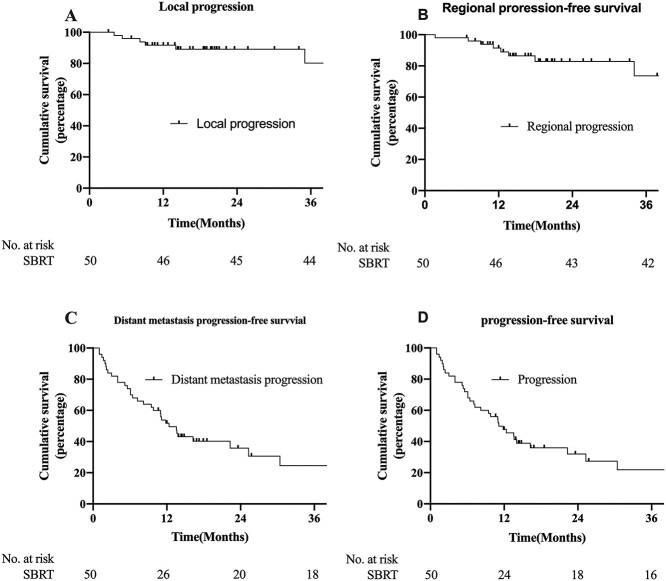
LPFS (A), RPFS (B), DMPFS (C) and PFS (D) of patients after SBRT for lung oligo-recurrence of NSCLC.

The median follow-up period was 23.6 (range, 3.1–61.7) months. A total of 36 (72%) patients developed disease progression before the final analysis. The lung oligo-recurrence of six (12%) of these patients developed LP after SBRT. The 1-year local progression-free survival (LPFS) was 91.7%, and the 2- and 3-year LPFS was 89.1% and 80.2%, respectively (Fig. 1A). Eight (16%) patients developed RP during follow-up; the corresponding 1-, 2- and 3-year rates of regional progression-free survival (RPFS) were 91.5%, 82.8% and 73.6%, respectively (Fig. 1B). However, DMP was the main reason for disease progression. In total, 33 (66%) patients in the entire group developed DMP. The rates of 1-, 2- and 3-year distant metastasis progression-free survival (DMPFS) were 51.7%, 35.8% and 24.5%, respectively (Fig. 1C). Overall, the median PFS was 11.1 months (95% CI: 7.0–15.3). The rates of 1-, 2- and 3-year PFS after SBRT were 47.7%, 31.9% and 21.9%, respectively (Fig. 1D).

**Fig 2:**
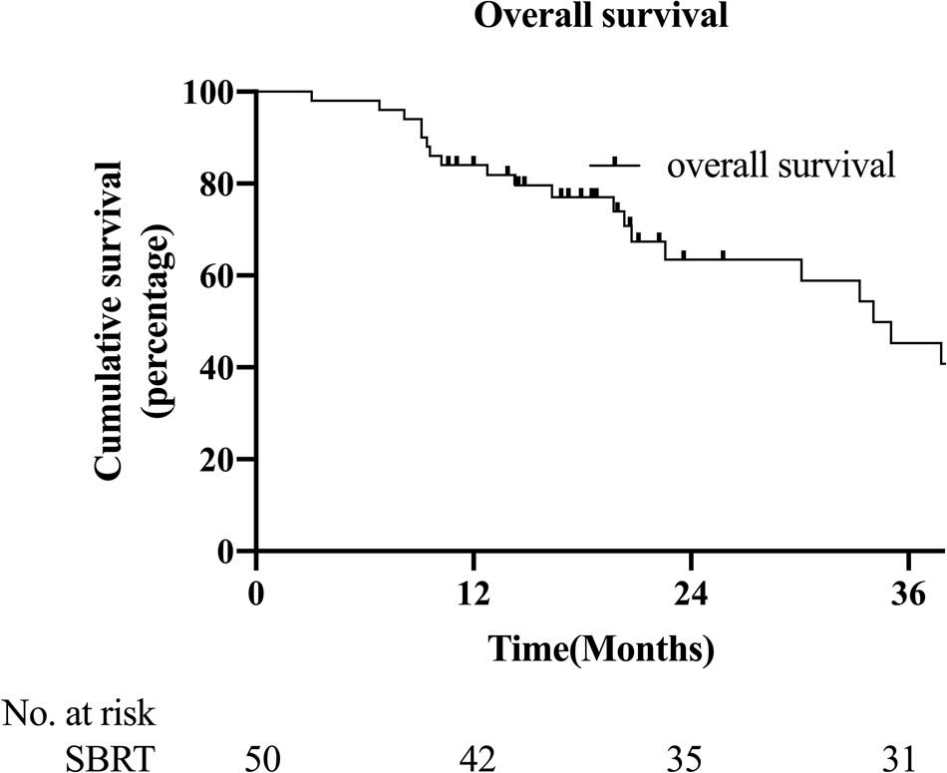
Kaplan–Meier curves and estimated cumulative incidences of OS after SBRT for lung oligo-recurrence of NSCLC.

The median OS was 34.1 months (95% CI: 26.3–41.9). A total of 21 (42%) patients in the entire group died during the follow-up period, all from lung cancer. The rates of 1-, 2- and 3-year OS after SBRT were 84%, 63.4% and 45.3%, respectively (Fig. 2).

**Fig. 3:**
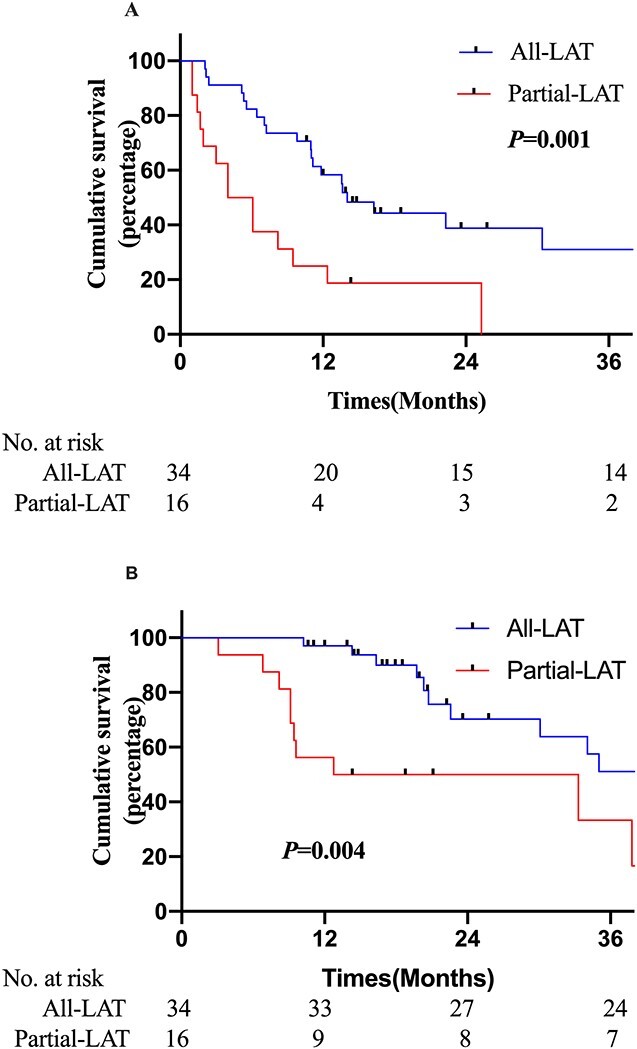
Kaplan–Meier plot of (A) PFS and (B) OS in all-LAT and partial-LAT subgroups.

To further clarify the benefit of consolidative LAT, patients were subdivided into two subgroups, namely all-LAT and partial-LAT. Patients in the all-LAT subgroup showed significantly improved PFS; these patients underwent consolidative LAT to primary tumor sites and all metastatic sites (*P* = 0.001; Fig. 3A). Meanwhile, compared with patients in the partial-LAT subgroup, those in the all-LAT subgroup showed significantly better OS (*P* = 0.004; Fig. 3B).

### Univariate and multivariate analysis of PFS and OS

Univariate analysis revealed that female sex, isolated recurrence site and all-LAT were significantly associated with better PFS. Multivariate analysis revealed that all-LAT was an independent predictor of better PFS (HR = 0.348, 95% CI: 0.171 – 0.708, *P* = 0.004; Table 3). Univariate analysis of OS showed that female sex, small size of the lung oligo-recurrence site, isolated recurrence site and all-LAT were associated with better OS. Multivariate analysis identified all-LAT as an independent predictive factor of better OS (HR = 0.315, 95% CI: 0.124 – 0.802, *P* = 0.015; Table 4).

**Table 3:** Univariable and multivariable analysis of covariables associated with progression-free survival

Variable	Univariable Analysis			Multivariable Analysis		
	HR	95% CI	*P*	HR	95% CI	*P*
Age (y)						
<65 vs ≥65	1.325	0.682–2.573	0.407			
Gender						
Female vs Male	0.395	0.171–0.913	0.030	2.447	1.052–5.690	0.038
ECOG PS						
0–1 vs 2	0.840	0.323–2.180	0.720			
Histology						
Adenocarcinoma vs Non-adenocarcinoma	0.818	0.424–1.576	0.548			
EGFR mutations						
Yes vs No	0.866	0.410–1.832	0.707			
Stage						
I–III vs IV	0.944	0.449–1.986	0.880			
Diameter of lung metastases						
<2 cm vs ≥2 cm	0.607	0.299–1.229	0.165			
Number of metastases						
1 site vs >1 sites	0.414	0.206–0.832	0.013	1.955	0.962–3.972	0.064
Combined with chemotherapy or TKI						
Yes vs No	0.736	0.351–1.543	0.417			
LAT						
All LAT vs Partial LAT	0.335	0.166–0.676	0.002	0.348	0.171–0.708	0.004

PFS, progression-free survival; HR, hazard ratio; CI, confidence interval; ECOG, Eastern Cooperative Oncology Group; PS, performance status; LAT, local ablative therapy; EGFR, epidermal growth factor receptor; TKI, tyrosine kinase inhibitor

**Table 4:** Univariable and multivariable analysis of covariables associated with OS

Variable	Univariable Analysis			Multivariable Analysis		
	HR	95% CI	*P*	HR	95% CI	*P*
Age (y)						
<65 vs ≥65	0.793	0.332–1.893	0.601			
Gender						
Male vs Female	0.295	0.097–0.898	0.032	2.451	0.772–7.785	0.128
ECOG PS						
0–1 vs 2	0.451	0.165–1.237	0.122			
Histology						
Adenocarcinoma vs Non-adenocarcinoma	0.913	0.386–2.159	0.836			
EGFR mutations						
Yes vs No	0.745	0.272–2.044	0.568			
Stage						
I–III vs IV	0.893	0.325–2.457	0.827			
Diameter of lung metastases						
<2 cm vs ≥2 cm	0.362	0.153–0.859	0.021	1.724	0.679–4.376	0.252
Number of metastases						
1 site vs >1 sites	0.381	0.156–0.929	0.034	2.481	0.952–6.465	0.063
Combined chemotherapy or TKI treatment						
Yes vs No	0.507	0.169–1.517	0.224			
LAT						
All LAT vs Partial LAT	0.296	0.122–0.715	0.007	0.315	0.124–0.802	0.015

OS, overall survival; HR, hazard ratio; CI, confidence interval; ECOG, Eastern Cooperative Oncology Group; PS, performance status; LAT, local ablative therapy; EGFR, epidermal growth factor receptor; TKI, tyrosine kinase inhibitor

### Toxicity

No treatment-related death was encountered in the entire group. During the entire follow-up period, the majority of adverse events due to SBRT were pneumonitis, including grade 1 pneumonitis in 15 (30%) patients, grade 2 in nine (18%) patients, and grade 3 in four (8%) patients. All the four patients who experienced grade 3 pneumonitis had multiple (≥2) lung oligo-recurrence sites. Other mild (grade 1–2) complications were observed in 18 (36%) patients: nine (18%) patients developed a cough, 4 (8%) patients experienced radiation esophagitis, three (6%) experienced dyspnea and one (2%) experienced chest pain (Table 5).

**Table 5:** Toxicity in patients

Toxicity	Patient No. (%)
Pneumonitis Grade 1 Grade 2 Grade 3	15 (30) 9 (18) 4 (8)
Chest pain	1 (2)
Fatigue	1 (2)
Esophagitis	4 (8)
Dyspnea	3 (6)
Cough	9 (18)

## DISCUSSION

Recently, certain patients with oligo-recurrence have been founded to achieve long-term survival and even get cured by definitive local therapy alone [13, 14]. For lung oligo-recurrence, surgery is traditionally considered to be the first treatment choice. However, in some cases, SBRT, as a minimally invasive definitive local therapy, may be better suited to patients with high operation risk or for those who refuse surgery. Through this study, we aimed to assess the outcomes of SBRT for lung oligo-recurrence of NSCLC.

The primary goal of curative SBRT in the study was to maximize local tumor control. Existing data highlight that a minimum of 100 Gy BED_10_ improved the LPFS of patients with lung metastasis [15–17]. Hence, we used a similar higher BED for all lung oligo-recurrence. This retrospective study showed an excellent LPFS of 80.2% at 3 years, which was consistent with previously reported data on SBRT and surgery for lung metastases [18–21]. However, few studies have directly compared LPFS between SBRT and surgery. Widder *et al.* reported that the 3-year LPFS for lung oligo-recurrence from various cancers was 85% after SBRT and 83% after metastasectomy [20]. Lodeweges *et al.* conducted a 5-year follow-up of surgery or SBRT for lung oligo-recurrence and found that SBRT is comparable to surgery on in terms of LPFS (83% vs 81%) [21]. Further, the cumulative incidence of RP in our study was only 16% at 3 years, with the corresponding 1-, 2- and 3-year RPFS being 91.5%, 82.8% and 73.6%, respectively. The RPFS was comparable to the reported data of early-stage NSCLC treated with SBRT or surgery [22, 23]. Also, in our cohort, SBRT yielded 1-, 2- and 3-year OS rates of 84%, 63.4% and 45.3%, respectively. These OS data were consistent with other SBRT data. A recent retrospective study investigated a total of 1378 patients with 1547 pulmonary oligometastases treated with SBRT, among whom 1016 patients developed lung oligo-recurrence. With a median follow-up period of 24.2 months, the 1- and 3-year OS rates were 90.1% and 60.3%, respectively [24]. Moreover, a phase II clinical trial at the MD Anderson Cancer Center enrolled 59 patients who developed isolated lung oligo-recurrence following definitive treatment of stage I–III NSCLC. After SBRT, the rate of 5-year OS in these patients was 56.5%, and the median OS was 63.8 months [13]. However, to the best of our knowledge, few studies have compared OS data of SBRT and metastasectomy. In 2013, Widder *et al.* first compared pulmonary oligo-recurrence resection with SBRT in patients with controlled primary tumor sites. A median follow-up of 43 months showed no significant difference in OS between surgery and SBRT (3-year OS: 62% vs 60%, respectively) [20]. In 2017, Lodeweges and Widder *et al.* furtherly analysed the clinical outcomes of patients with lung oligo-recurrence from various primary tumors who underwent SBRT or metastasectomy. The 5-year OS was not significant between patients who underwent SBRT and those who underwent surgery (45% and 41%, respectively) before as well as after propensity score matching [21]. Moreover, Londero *et al.* recently conducted a systematic review comparing SBRT with surgery for pulmonary metastases. The patient population in the selected 79 original articles contained patients with oligo-recurrence and other oiligometastatic lung sites. The investigation showed that SBRT and surgery had similar survival outcomes in the short term; however, less toxicities were reported for SBRT, while surgery might have better survival rates in the long term. The authors found that larger populations and longer follow-up times in the surgery group rendered the results from surgical reports more reliable [25]. However, there is no evidence from prospective clinical tials regarding the comparison between SBRT and surgery in patients with lung oligo-recurrence. Therefore, when choosing the optimal local treatment, patients selection seems to play a crucial role. The decision should weigh not only the potential locoregional control and survival benefit but also the risks of toxicity profile. Surgery also has an advantage of acquiring tissues for pathologic evaluation of treatment response characteristics, which can used to develop subsequent therapeutic strategies such as immunotherapy or targeted therapy. SBRT might induce not only double-strand breakage of turmor cell, but also activation of immume function of the tumor microenvironment [26]. Consequently, some authors consider SBRT as an available alternative to surgery for patients with lung oligo-recurrence [27].

However, SBRT for the limited recurrence disease has demonstrated survival advantages over standard-of-care therapy in several randomized studies [28, 29]. Ost *et al*. conducted a prospective randomized multicenter phase II study on patients with oligo-recurrence prostate cancer, in which SBRT was compared with surveillance. The study revealed that androgen deprivation therapy-free survival was longer with SBRT than with surveillance alone [29]. Notably, Palma *et al*. reported regarding the randomized phase II SABR-COMET trial, in which 99 patients with controlled cancer with 1-5 oligo-recurrence metastatic lesions underwent SBRT. After a median follow-up of 25 months, the SBRT group achieved significantly better median OS than the standard care group did (41 months and 28 months, respectively) [28].

In our study, DMP was still the main pattern of failure in our study population. Thirty-three (66%) patients developed DMP, and the 3-year DMPFS was 24.5%. A multicenter randomized phase II trial investigated the clinical results for oligo-recurrence by SBRT and reported that 79% of patients developed new lesions [28]. The high DMP rate after SBRT reflected the underlying tumor biology of aggressive and recurrent disease. The timing of these relapses suggests that these patients had developed microscopic pre-existing distant seeding at the time of SBRT. Therefore, patients with lung oligo-recurrence who receive only local treatment should be closely monitored. It is hinted that many patients might benefit from concurrent systemic treatment at the time of recurrence. A retrospective study of 57 patients with locoregionally recurrent NSCLC after surgery suggested that the use of concurrent chemotherapy was associated with better survival than radiotherapy alone was [30]. However, immune checkpoint inhibitors have reported varied success in locally advanced and metastatic NSCLC in the past few years, and are better tolerated than chemotherapy [31]. Tang *et al.* designed the first phase I–II prosepctive trial combining SBRT with immunotherapy. SBRT was concurrently or sequentially delivered with ipilimumab to liver or lung metastases. In phase I, 7/31 (23%) patients experienced partial response or stable disease lasting more than 6 months outside the radiation field [32]. In phase II, 106 patients was enrolled. The median PFS was 2.9 months after a median follow-up of 10.9 months. Among them, 67% patients with NSCLC had partial response or stable disease [33]. Luke *et al.* conducted a phase I study of pembrolizumab with multisite SBRT in patients with metastatic solid tumors. In the study, all 79 patients were heavily pre-treated. After a median follow-up of 5.5 months, the overall objective rate was 13.2% and the median OS and PFS were 9.6 and 3.1 months, respectively. Notably, the abscopal response rate was 13.5% [34]. Recently, the PEMBRO-RT trial, a phase II randomized clinical trial, evaluated the combination of SBRT and immunotherapy in patients with recurrent metastatic NSCLC. The overall response rate of the experimental arm and the control arm was 36% and 18% at 12 weeks (*P* = 0.07). The study found improved PFS in PD-L1 negative patients who received SBRT followed by pembrolizumab, in comparion to those who received pembrolizumab alone [35]. These clinical trials involving a combination of SBRT with immunotherapy was well tolerated and showed promising results. However, it is still difficult to confirm what kind of systemic therapy would improve the survival outcome and in which subset of patients.

In the process of determining which patients will achieve better outcomes with SBRT, we found that several clinical factors were associated with survival outcomes. Among them, Female sex and isolated recurrence site were significantly associated with PFS and OS in our study, which is consistent with other data [36, 37]. These two factors were speculated to correlate with slow-growing nature of the tumor [3, 37]. And the median recurrence interval was 19.7 months in the present study, which is another proof for the slow development of recurrence. Notably, patients who underwent all-LAT had better survival outcomes than those who underwent partial-LAT did. Few studies have reported the importance of all residual diseases being treated by consolidative therapy. However, Xu *et al*. reported that all-LAT can prolong both PFS and OS compared with partial-LAT in patients with synchronous oligometastatic stage IV EGFR-mutant NSCLC [38]. Further, after a 5-year follow-up of 59 patients, the authors from MD Anderson Cancer Center concluded that all-LAT to lung oligo-recurrence is an essential step to obtain a cure or a better prognosis [13]. Notably, the only phase II randomized clinical trial, the SABR-COMET trial, found that all-LAT (SBRT) was associated with an improvement in survival outcomes [28]. Thus, LAT might be a potiential cure for all residual diseases in patients with NSCLC who show slow development of recurrence. However, the clinical factors require further validation in future research.

With regard to treatment-related toxicity, our findings showed that SBRT was well tolerated and had a low incidence of toxicity profile; only 8% of patients experienced grade 3 adverse events. Compared with surgery, SBRT was associated with less severe treatment-related complications and death [7]. All the grade 3 adverse events in the study involved radiation pneumonitis. However, our result sugguested that grade 3 pneumonitis was closely related to muliple (≥2) lung oligo-recurrence sites. The other mild toxicities included grade 1–2 pneumonitis, cough, radiation esophagitis, dyspnea and chest pain. The low incidence of toxicity in our study could be attributed to the small tumor size and peripheral location of most lung oligo-recurrence.

This study has several limitations. First, this retrospecctive study was conducted at a single institution with small sample of enrolled patients, which would have had a degree of intrinisic bias. Also, the follow-up period of the patients with SBRT was short, which may have affected their survival rates. Second, fewer patients underwent histological biopsy to confirm NSCLC oligo-recurrence. Although multi-modality diagnostic imaging, including contrast-enhanced CT scans, PET-CT and MRI of the brain, was used for evaluation of the lung lesions, a definite clinical diagnosis of recurrence without pathological examination was not sufficient. Therefore, some of the lung oligo-recurrence might be metachronous second primary lung cancers. This inaccuracy is a crucial flaw of this study. Moreover, fewer patients underwent a pretreatment PET-CT scan, which may have influenced the judgment about whether the DM developed before or after SBRT. Finally, systemic therapy, including traditional chemotherapy, EGFR-TKI and checkpoint immunotherapy, should also be taken into consideration in further studies.

## CONCLUSION

In conclusion, SBRT for lung oligo-recurrence of NSCLC showed excellent local control, low toxicity and promising survival rates during a short-term follow-up. In addition, LAT to all residual diseases may be associated with better survival outcomes in patients with slow development of recurrence. More data are needed to define the optimal patient selection for SBRT for lung oligo-recurrence of NSCLC. Importantly, most patients developed distant metastasis after SBRT. Based on ongoing clinical trails, it is clear that the use of concurrent systemic therapy or immunotherapy may offer survival benefits [39]. SBRT could be considered as an effective and safe strategy to treat patients with lung oligo-recurrence of NSCLC. However, more evidence from further studies, especially large population-based prospective studies, is warranted.
